# GC3558: An open-source annotated dataset of Ghana currency images for classification modeling

**DOI:** 10.1016/j.dib.2022.108616

**Published:** 2022-09-17

**Authors:** Kwabena Adu, Patrick Kwabena Mensah, Mighty Abra Ayidzoe, Obed Appiah, Ebenezer Quayson, Christopher Bombie Ninfaakang, Michael Opoku

**Affiliations:** Department of Computer Science and Informatics, University of Energy and Natural Resources, Sunyani, Ghana

**Keywords:** Dataset, Deep learning, Classification, Currency detection, Banknote recognition

## Abstract

The field of deep learning has led to remarkable advancements in many areas, including banking. Identifying currency denomination type and model is challenging due to intraclass variation and different illumination conditions. Although, in this domain, many datasets regarding currency denomination type and model, e.g., Indian Currency, Thai Currency, Chinese Currency, U.K. currency, etc., have already been experimented with by different researchers. More datasets are needed from a variety of currencies, especially Ghana currency (cedi). This article presents the Ghana Currency image dataset (GC3558) of 3558 color images in 13 classes created from a high-resolution camera. The dataset is comprised of only genuine currency. The class consists of coin and paper notes: 10 pesewas coin, 20 pesewas coin, 50 pesewas coin, 1 cedi coin, 2 cedis coin, 1 cedi note, 2 cedis note, 5 cedis note, 10 cedis note, 20 cedis note, 50 cedis note, 100 cedis note and 200 cedis note. All images are de-identified, validated, and freely available for download to A.I. researchers. The dataset will help researchers evaluate their machine learning models on real-world data.

## Specifications Table

**Table:** 

Subject	Machine Learning / Deep Learning
Specific subject area	Currency detection and identification
Type of data	Ghana currency images
How the data were acquired	The Ghana currency images were collected by taking images using a high-resolution camera device. Table 1 shows a description of the camera used to collect the dataset.
Data format	Raw Annotated
Parameters for the data collection	The Ghana currency dataset images are .jpg images of 1512×2016 dimension, and resolution is 96 dpi
Description of data collection	The denominations of the Ghana currency were collected using a high-resolution camera device. The original .jpg images of currency were in varied dimensions (1512×2016), (1560×2080), (2080×1560), and (1080×1440). These images are resized to 128×128 dimensions. There are total 13 classes of the Ghana currency namely 10_pesewas_coin, 20_pesewas_coin, 50_pesewas_coin, 1_cedi_coin, 2_cedi_coin, 1_cedi_note, 2_cedi_note, 5_cedi_note, 10_cedi_note, 20_cedi_note, 50_cedi_note, 100_cedi_note, and 200_cedi_note. The images were captured from various environmental conditions like white background, dark background, yellow background, and illuminated background.
Data source location	University of Energy and Natural Resources P.O. Box 214, Sunyani – Ghana
Data accessibility	Repository name: Dataset of Ghana Currency with Annotations Data identification number(doi): 10.17632/vws5r8mj4w Direct URL to data: https://data.mendeley.com/datasets/vws5r8mj4w/draft?a=42fcc651-6826-49f0-b20c-6adb0f632a91

## Value of the Data

• The dataset is comprehensive and consists of 3558 high-quality images of 13 different classes.
• The dataset consists of coins and paper notes denomination of the Ghana Currency.
• This dataset is useful for building applications for Ghana Currency classification and detection. It can also be used by researchers working in currency classification and identification.
• This dataset is useful for training, testing, and validating Ghana Currency or for classification and identification models.
• The dataset will play an important role in the value identification of Ghana Currency.
• The dataset will help build an application for currency classification, identification, and detection that can be used by visually impaired people, bank customers, governments, and various agencies.

## 1 Data Description

The currency dataset's creation is vital for the following reasons: Correct recognition of currency denomination is an essential task for automated teller machines (ATMs) and currency identification machines [1,2]. In addition, it is necessary to design a system that detects a genuine currency [3]. Furthermore, recognizing currency denominations is a problem for visually impaired people [4,5]. The dataset associated with this paper contains 3558 color images and consists of thirteen (13) classes. The original captured were in varied sizes of (1512×2016), (1560×2080), (2080×1560), and (1080×1440).

**Fig. 1:**
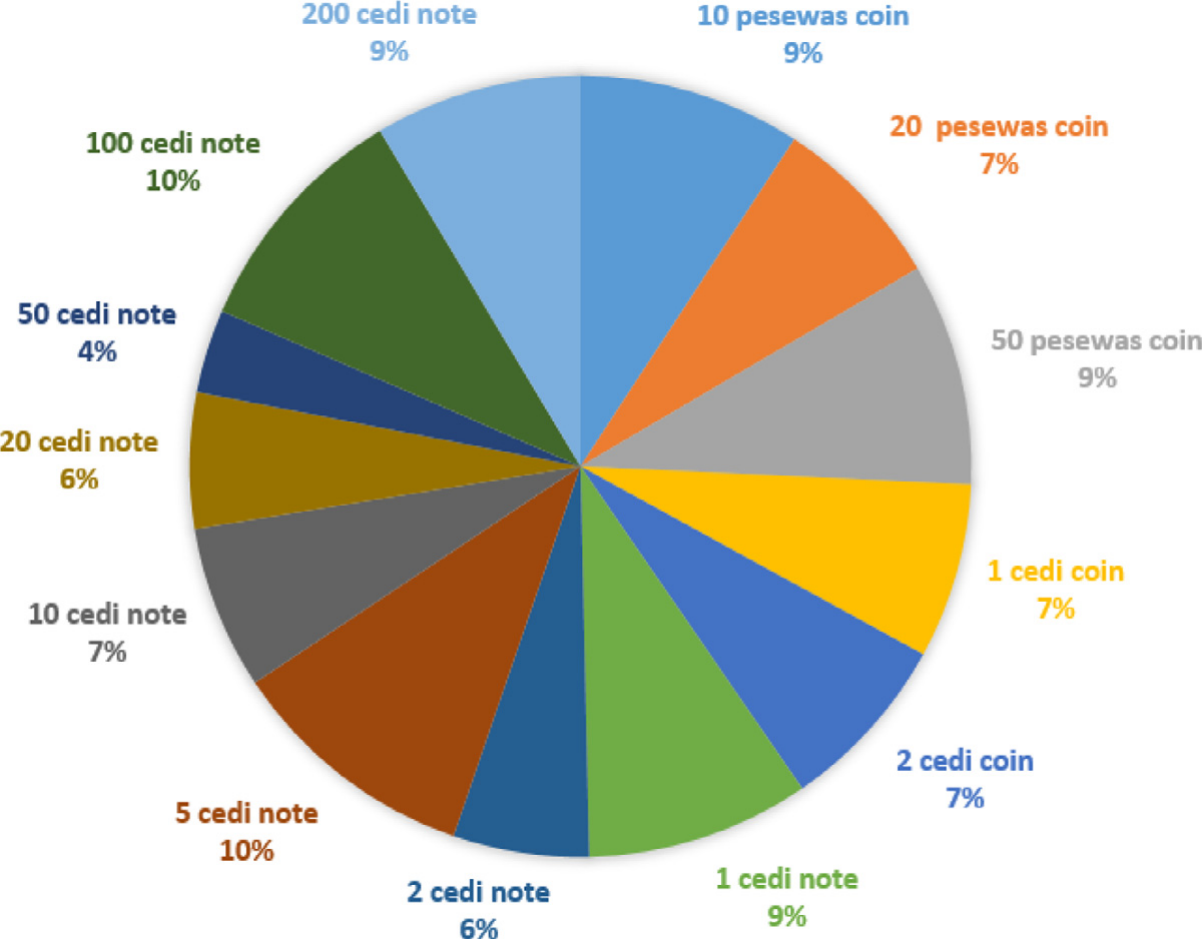
Percentage of each currency denomination in the GC3558 dataset.

This paper considers deep learning classification tasks on single and multiple models and input image resolution or size. Increasing image resolution for training with deep learning models often has a trade-off with the maximum possible batch size. Yet, the optimal selection of image resolution can further increase neural network performance for various image processing tasks [6]. Since the originally captured images were in varied resolutions, such as (1512×2016), (1560×2080), (2080×1560), and (1080×1440), and hence very large for training with deep learning techniques. Moreover, large input image sizes introduce memory constraints. As a result, there is an intense computational complexity and requirement, which leads to long training and inference time of the deep learning models [7]. For example, the training time on computer hardware with Graphical Processing Units for 2080 × 2080 pixels input images may take approximately 40 days of consecutive model training, which can be seen as impractical. To alleviate this problem of the time budget of training, we downscaled the image size to a dimension of 128×128. The downscaled 128×128 image pixels are in jpg file format. The dataset can be downloaded as a 1.98 GB zip file GC3558.zip. After unzipping, the main folder Ghana_Cedis Currency contains the Ghana Cedis Currency folder, which contains two subfolders: train and validation folder. Each of the two folders contains thirteen subfolders. The subfolders are 10_pesewas_coin (328), 20_pesewas_coin (261), 50_pesewas_coin (327), 1_cedi_coin (257), 2_cedi_coin (264), 1_cedi_note (329), 2_cedi_note (200), 5_cedi_note (370), 10_cedi_note (241), 20_cedi_note (200), 50_cedi_note (123), 100_cedi_note (353, and 200_cedi_note (305). Table 1 presents the camera specification used to capture the dataset. The resolution quality of the image dataset depends on the quality of the camera used. Therefore, the camera specification presented in Table 1 was used in capturing the GC3558 dataset. Table 2 shows the description of the dataset. The Table shows the various denominations, the direction of image capturing, backgrounds, and the number of images of each denomination. Fig. 1 illustrates the percentage of each denomination presented in the dataset. The Fig. 1 shows that the 5 cedi and 100 cedi notes contain 10% each, which is the highest representation of the total dataset. The 10pesewas coin, 50pesewas coin, 1 cedi, and 200 cedi notes comprise 9% each of the total dataset. The 20pesewas coin, 1 cedi coin, 2 cedi coin, and 10 cedi note comprise 7% each of the total dataset. The 2 cedi and 20 cedi notes comprise 6% each of the total dataset. The 50 cedi denomination comprises 4% of the total dataset, which is the least representation.

**Table 1:** Camera specifications.

	Description
Camera Name	Nikon D3500
Type	DSLR
Sensor	APS-C
Megapixels	24.2MP
Lens Mount	Nikon F
Videofinder	Optical
Max View Resolution	Full HD

**Table 2:** Description of Ghana Currency (GC3558) dataset.

S.N.	Denomination Considered	Direction of image Capturing	Different Backgrounds considered for image capturing	No. of Images of each denomination
1	10 pesewas coin	Front Direction, Front Direction Rotated 1800, Backward Direction, Backward Direction Rotated 1800	white, dark, yellow, and illuminated.	328
2	20 pesewas coin			261
3	50 pesewas coin			327
4	1 cedi coin			257
5	2 cedi coin			264
6	1 cedi note			329
7	2 cedi note			200
8	5 cedi note			370
9	10 cedi note			241
10	20 cedi note			200
11	50 cedi note			123
12	100 cedi note			353
13	200 cedi note			305
	**Total No. of Images**	**3558**

**Fig. 2:**
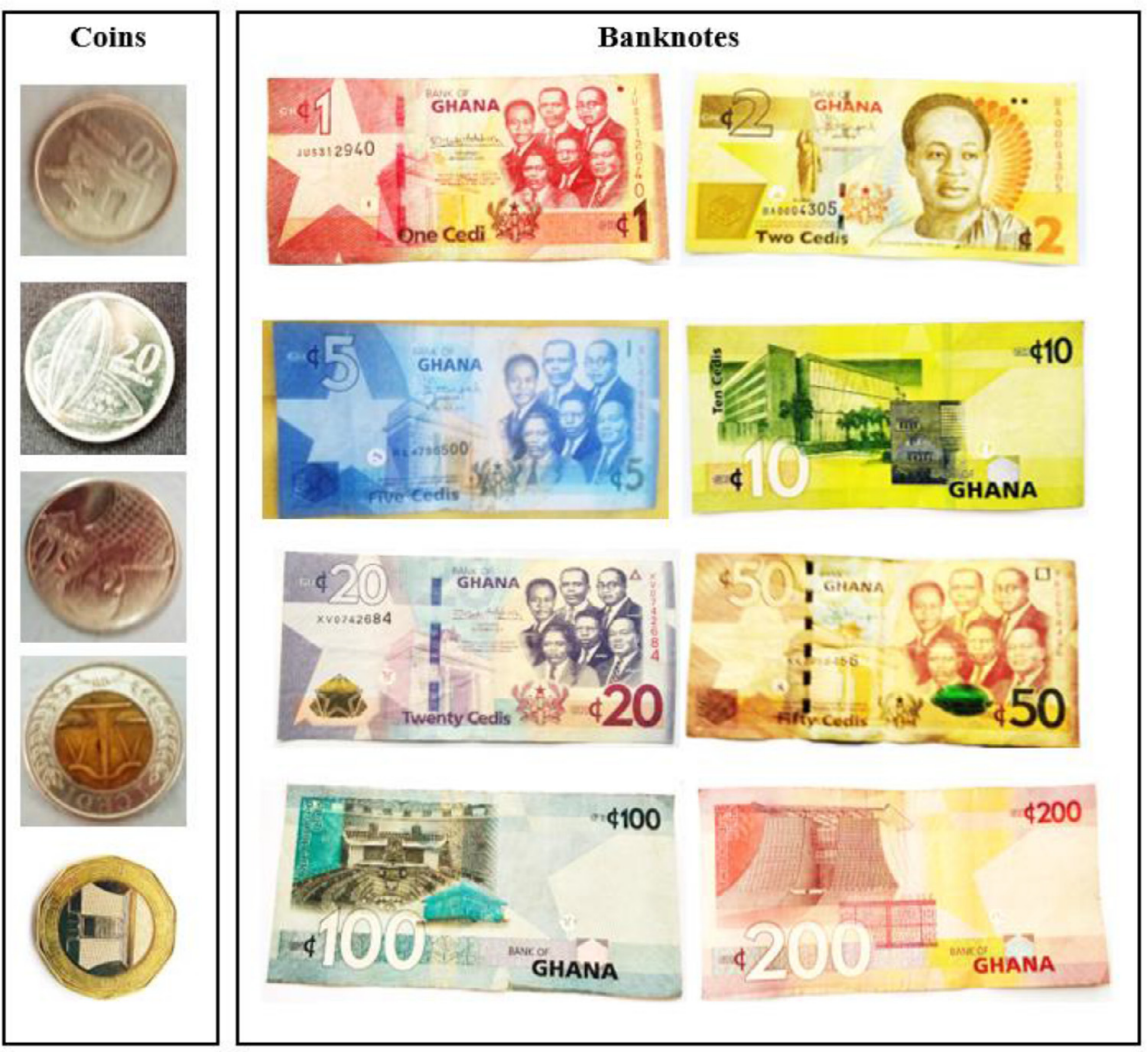
Data Samples of the GC3558 images.

**Fig. 3:**
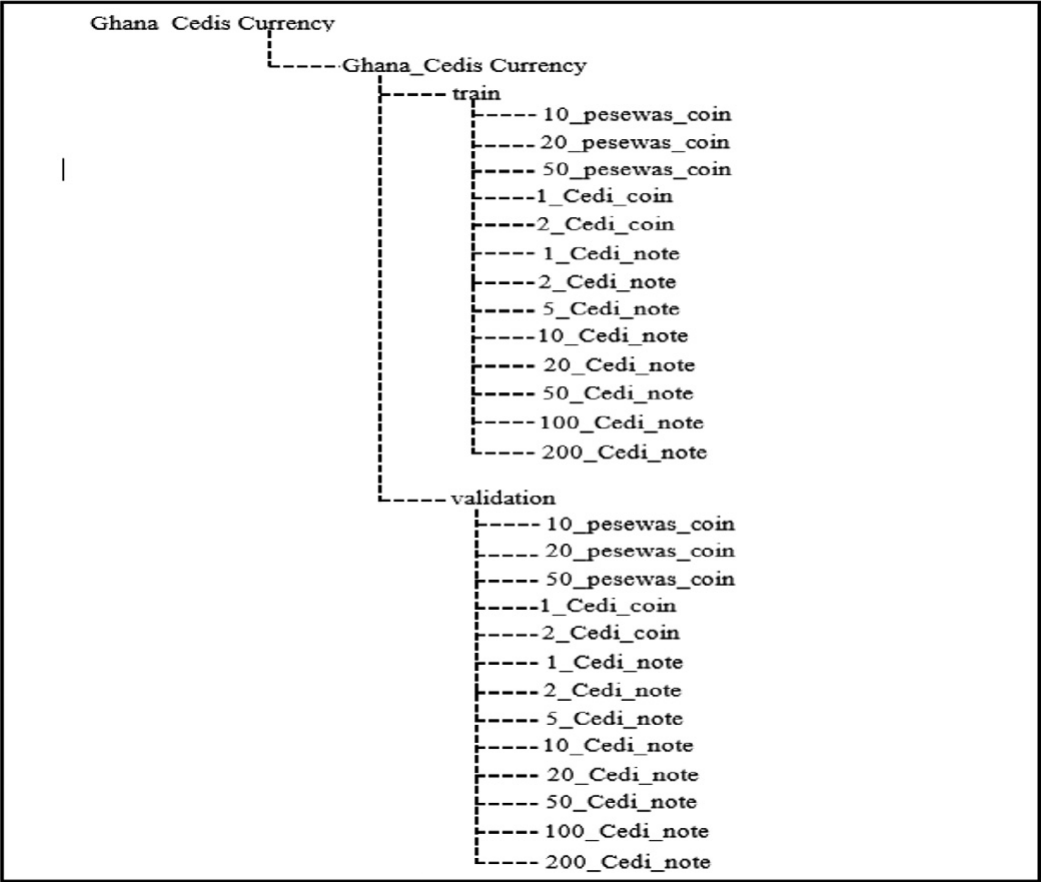
Ghana Currency dataset directory structure.

Fig. 2 shows data samples of the GC3558 images presenting the various currency denomination. The Figure shows both the coins (left) and banknotes (paper note) currencies (right). The directory structure of the currency dataset is shown in Fig. 3. Fig. 3 describes the folder structure of the GC3558 dataset. The first folder is Ghana Cedi Currency which contains a subfolder named Ghana Cedi Currency. In the subfolder, there are two (2) additional subfolders; train and validation, which contains the 13 classes of the Ghana currency images.

## 2 Experimental Design, Materials and Methods

### 2.1 Experimental design

**Fig. 4:**
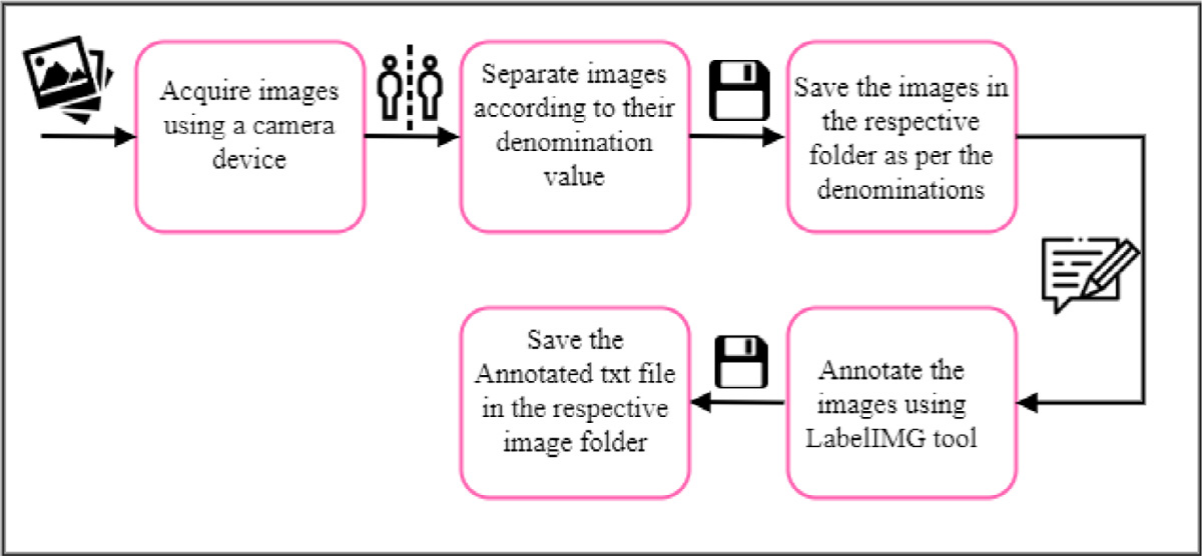
Ghana Cedis Currency (GC3558) dataset acquisition process.

Fig. 4 illustrates the image data acquisition process. The images were captured using Nikon D3500 high-resolution rear camera. All images were captured using a camera and then separated and saved in their respective folders per their denomination values. The images were annotated using labelIMG tool the annotated txt file was saved in a respective folder.

Table 3 gives a detailed description of the dataset acquisition process, and a description of the cameras is specified in Table 1. The Ghana Cedis Currency (GC3558) images were captured daily and during day time from November 2021 to January 2022. The images were captured in different directions and backgrounds and with variant sizes, as mentioned in Table 2. After the captured images were further separated into specific folders. The folder structure of images is shown in Fig. 3. The images were resized to 128×128 dimensions using python script and then annotated using the LabelImg tool from 2022 February to April 2022. The dataset is comprised of only genuine currency. Therefore, the authors have planned to update the dataset with counterfeit currencies in the future version, which is believed to help further improve the identification of genuine and counterfeit currency.

**Table 3:** Data acquisition steps.

No.	Step	Duration	Activity
1	Data Gathering	November 2021 to January 2022	Daily and during daytime capturing of the currency images
2	Image Labeling	February 2022 to April 2022	Labeled the 3558 images of Ghana Cedis Currency images

### 2.2 Materials or specifications of the image acquisition system

The Ghana currency images were captured using Nikon D3500 with a rear camera of 24.2 MP. All the original image datasets were of varied sizes (1512×2016), (1560×2080), (2080×1560), and (1080×1440) and were resized to 128×128 dimensions using a python script. The images were saved in .jpg format.

Robots perceive objects effectively and efficiently, highlighting the need to understand the environmental factors and their impact on visual perception, such as illumination changes. In object recognition and classification, illumination of the scene and differences in sensor capturing are two factors [8]. The inconsistency of quality between the training and testing images reduces the performance of deep learning models. Furthermore, inconsistency in lighting also reduces deep learning performance; however, introducing different lighting conditions can alleviate the reduction of performance [9].

In this paper, the images are captured in various environmental conditions such as different light conditions, different backgrounds, and from different angles. Capturing the images in these conditions serve as data augmentation, where more data is generated to train the deep learning model. Additionally, this technique helps to achieve better generalizability and improve the robustness of the deep learning model.

After capturing the images, they were organized as Ghana Cedis Currency. The Ghana currency dataset consists of 13 different folders. The dataset directory structure of images is shown in Fig. 3. The images are annotated using the LabelImg tool. The annotations images of currency are stored in their respective folders.

### 2.3 Method

The images were acquired using the Nikon D3500 camera in different angles and backgrounds. The original images were of different varied sizes (1512×2016), (1560×2080), (2080×1560), and (1080×1440) and were resized to 128 × 128 using a python script and then labeled using the LabelImg tool. Table 2. describes the classes, number of images, and the environments in which images were taken.

## Ethics Statement

There is no funding present for the present effort. There is no conflict of interest. The data is available in the public domain.

## CRediT Author Statement

**Kwabena Adu:** Methodology, Software, Writing – original draft; **Patrick Kwabena Mensah:** Data curation, Conceptualization, Supervision; **Mighty Abra Ayidzoe:** Writing – review & editing; **Obed Appiah:** Software, Validation; **Ebenezer Quayson, Christopher Bombie Ninfaakang** and **Michael Opoku:** Data curation, Investigation.

## Declaration of Competing Interest

The authors declare that there is no conflict of interest regarding the publication of this paper.

## Data Availability

An Open-Source Annotated Dataset of Ghana Currency Images (Original data) (Mendeley Data).
